# Gut microbial bile salt hydrolase as a metabolic gatekeeper in digestive homeostasis and disease

**DOI:** 10.3389/fimmu.2026.1870619

**Published:** 2026-06-09

**Authors:** Haitao Chu, Xin Song, Chang Liu, Tianjiao Ma, Wanlin Cui

**Affiliations:** 1 Department of Hyperbaric Oxygen Medicine, The First Hospital of China Medical University, Shenyang, Liaoning, China; 2 Department of Pharmacy, Guang’anmen Hospital, China Academy of Chinese Medical Sciences, Beijing, China; 3 Department of Pediatrics, The First Hospital of China Medical University, Shenyang, Liaoning, China

**Keywords:** bile acid metabolism, BSH, digestive diseases, gut microbiota, gut–liver axis, microbiota-based therapy

## Abstract

The gut microbiota exerts broad control over host physiology through tightly coordinated metabolic networks. Among these, bile salt hydrolase (BSH), a microbial enzyme, serves as a key mediator of microbiota–host crosstalk. As a key upstream gateway reaction in bile acid metabolism, BSH hydrolyzes conjugated bile acids and reshapes the composition and distribution of the intestinal bile acid pool. This remodeling alters bile acid signaling quality and influences the host energy metabolism, immune homeostasis and intestinal barrier integrity. In this review, we summarize the core biological functions and molecular regulatory mechanisms of microbial BSH in maintaining digestive system homeostasis. We further discuss the association between BSH dysregulation and the development of major digestive diseases, including metabolic dysfunction–associated steatotic liver disease (MASLD), inflammatory bowel disease (IBD) and colorectal cancer (CRC). Finally, we outline emerging precision strategies targeting BSH, including strain-specific probiotics, enzyme activity modulators and dietary interventions. These approaches offer a conceptual framework for microbiota-based therapies in digestive diseases.

## Graphical Abstract

**Figure:**
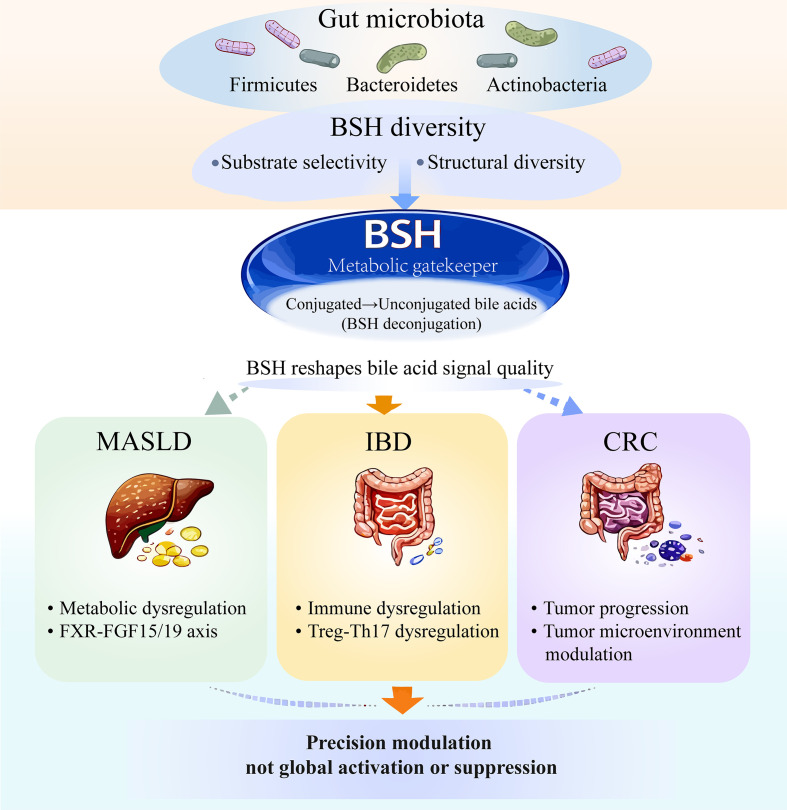
Context dependent metabolic gatekeeping by BSH in digestive diseases. BSH, widely distributed among gut microbiota including Firmicutes, Bacteroidetes and Actinobacteria, exhibits substantial diversity characterized by differences in substrate selectivity and structural variation. Acting as an upstream metabolic gatekeeper, BSH catalyzes bile acids deconjugation and reshapes bile acid signal quality, thereby influencing host signaling pathways. Through modulation of bile acid-receptor networks, altered BSH activity contributes to metabolic dysregulation in MASLD, immune dysregulation in IBD, and tumor microenvironment modulation in CRC. These context-dependent effects highlight the importance of precision modulation of BSH activity rather than global activation or suppression.

## 1 Introduction

Bile acids are cholesterol-derived molecules synthesized in the liver. They facilitate lipid absorption and act as signaling mediators that regulate metabolic and immune pathways. Maintaining bile acid homeostasis is therefore essential for proper gut–liver axis function (1, 2). Bile salt hydrolase (BSH) is widely distributed among gut bacteria. Differences in BSH activity markedly shape bile acid composition and affect host glucose and lipid metabolism, barrier integrity and immune homeostasis through farnesoid X receptor (FXR), G protein-coupled bile acid receptor 1 (also known as TGR5) and related pathways (3, 4). Within the microbial bile acid transformation network, BSH catalyzes the deconjugation of conjugated bile acids and is widely regarded as the gateway reaction of microbial bile acid metabolism (3, 5–7). This step alters the conjugated-to-unconjugated bile acid ratio and provides substrates for downstream transformations, including 7α-dehydroxylation and epimerization, thereby influencing secondary bile acid production and receptor signaling output.

Advances in metagenomics, metabolomics and germ-free animal models have clarified the functional landscape of BSH in digestive homeostasis and disease. These insights position BSH as a promising target for translational intervention. In metabolic dysfunction–associated steatotic liver disease (MASLD), alterations in BSH-associated pathways reshape bile acid composition and receptor signaling, driving systemic metabolic reprogramming and correlating closely with disease progression (8–10). In inflammatory bowel disease (IBD), impaired BSH function is closely linked to disrupted immune regulation. Clinical and functional studies have consistently reported reduced secondary bile acid production and the depletion of bile acid–transforming microbes in patients with IBD. These changes may disturb the regulatory T cells (Tregs)–T helper 17 cells (Th17 cells) balance and inflammasome activation, thereby sustaining mucosal inflammation and exacerbating disease activity (11–13). In tumors, BSH-mediated bile acid remodeling extends to the tumor microenvironment. In models of colorectal cancer (CRC) liver metastasis, the microbiota associated with reduced BSH activity alters bile acid–dependent immune signaling, promotes proinflammatory cell recruitment and creates a permissive metastatic niche (14, 15). BSH expression and activity are further shaped by strain specificity, substrate preference and host environmental factors, including diet, circadian rhythms and oxidative modifications (4, 16–18). Collectively, these findings indicate that BSH is not merely a metabolic enzyme but a regulatory node that links microbial function to host metabolism, immunity and tumor ecology. The precise modulation of BSH activity may therefore be critical for maintaining digestive homeostasis and for therapeutic intervention.

In this review, we outline the biological functions and regulatory mechanisms of BSH, examine its role in digestive diseases and discuss emerging strategies that target BSH for precision intervention. Unlike previous reviews that have broadly summarized bile acid–microbiota interactions, BSH enzymology or bile acid receptor signaling, this review specifically focuses on BSH as an upstream microbial metabolic gatekeeper that reshapes the quality, spatial distribution and immunological consequences of bile acid signals. We integrate recent findings on canonical BSH mediated deconjugation, emerging reconjugation, disease-specific bile acid remodeling and immune regulation across the gut–liver axis. This framework emphasizes that the biological effects of BSH are context-dependent rather than uniformly protective or harmful.

## 2 Microbial origin, structural diversity and functional heterogeneity of BSH

### 2.1 Phylogenetic distribution and enzymatic characteristics of BSH-producing bacteria

BSH exhibits a broad and conserved phylogenetic distribution within the gut microbiota. Genomic and metagenomic analyses have revealed that *bsh* genes are predominantly found in core phyla, including Firmicutes, Bacteroidetes and Actinobacteria (3, 19). At the genus level, multiple common commensals, including *Lactobacillus*, *Bifidobacterium*, *Clostridium* and *Bacteroides*, encode distinct *bsh* variants (4, 20–22). During early-life microbiota development, the abundance of BSH and BSH-related genes increases over time in parallel with enhanced bile acid transformation capacity (23). These observations suggest that BSH participates in shaping the bile acid signaling environment during the establishment of microbiota–host interactions.

Despite sharing a common catalytic function, BSH enzymes from different taxa display substantial variation in amino acid sequence, three-dimensional structure and substrate recognition mechanisms (24–26). Most BSH proteins belong to the N-terminal nucleophile hydrolase family and retain a conserved catalytic core. However, subtle differences within the substrate binding pocket markedly influence the preference for glycine- or taurine-conjugated bile acids (24, 27). On the basis of substrate selectivity, BSH can be classified into distinct functional subtypes, often reflecting ecological niche adaptation and metabolic strategy. Importantly, these enzymatic differences extend beyond molecular properties. Distinct BSH variants generate nonequivalent deconjugation patterns *in vivo*, potentially creating spatially and temporally distinct bile acid signaling inputs (20, 28). Amplification through bile acid receptor networks may then translate these differences into divergent metabolic or immune outcomes in the host (12, 13). Different bile acid species have distinct receptor preferences and immune-metabolic consequences. For example, chenodeoxycholic acid (CDCA) is a potent endogenous ligand for FXR, whereas the secondary bile acids deoxycholic acid (DCA) and lithocholic acid (LCA) can activate TGR5 (29, 30). In addition, BSH-mediated deconjugation provides unconjugated bile acid substrates for downstream microbial transformations, generating immunologically active derivatives (7, 13, 28). Microbially modified LCA derivatives, including 3-oxoLCA and isoalloLCA, have been linked to Th17 suppression and Treg induction, respectively (13, 31).

Recent technological advances have further expanded the functional landscape of BSH. The use of activity-based probes (ABPs) combined with chemical proteomics enables direct profiling of BSH catalytic activity within complex microbial communities (32, 33). Moreover, emerging evidence indicates that certain BSH variants exhibit noncanonical activities, including acyltransferase functions, and may participate in the generation of novel bile acid conjugates (18, 22). These findings broaden the conceptual framework describing BSH from a simple hydrolase to an active contributor to bile acid chemical diversification, with potential consequences for host signaling networks.

In addition to genetic and structural determinants, BSH catalytic efficiency is dynamically shaped by local environmental conditions. Optimal activity typically occurs within neutral to mildly alkaline pH ranges, which is consistent with the distal ileum and colon environment (34, 35). Ionic strength, substrate availability and bile acid composition further modulate enzymatic performance (36, 37). This environmental responsiveness enables BSH to rapidly adjust its functional output during physiological shifts or microbial community remodeling.

Collectively, the phylogenetic diversity, substrate selectivity and environmental adaptability of BSH define its unique position within the bile acid metabolic network. BSH therefore functions not only as a deconjugating enzyme but also as a regulatory interface that links microbial ecological dynamics with host signaling control.

### 2.2 Biochemical mechanism of BSH catalysis and remodeling of the bile acid pool

BSH hydrolyzes the amide bond of conjugated bile acids, generating unconjugated bile acids and releasing the corresponding amino acids (5, 38). This deconjugation reaction directly affects the conjugated-to-unconjugated bile acid ratio within the intestinal lumen and reshapes the substrate landscape for downstream microbial bile acid metabolism and receptor signaling. By altering the physicochemical properties of bile acids and their enterohepatic circulation dynamics, BSH-dependent deconjugation alters the exposure and distribution of bile acids species along the gut–liver axis. Moreover, deconjugation provides essential substrates for subsequent microbial transformations, including 7α-dehydroxylation, thereby influencing secondary bile acid profiles and receptor signaling output (2, 39, 40). Accordingly, the level of BSH activity largely determines the extent to which downstream metabolic networks are initiated or amplified. This regulatory effect extends beyond chemical structure alone. Deconjugation affects bile acid solubility, membrane permeability and circulation kinetics, shaping the spatial distribution and portal exposure of bile acid along the intestine (41). Differences in substrate availability further influence secondary bile acid production and reshape the overall composition of the bile acid pool (28, 42).

Emerging evidence indicates that BSH-mediated remodeling of the bile acid pool is highly context dependent. The consequences of deconjugation vary according to the microbial community structure, substrate availability and host physiological state. For example, in preterm neonates with cholestasis, reduced BSH activity is correlated with decreased levels of deconjugated bile acids, impaired bile acid circulation and metabolic disturbance (43, 44).

**Figure 1:**
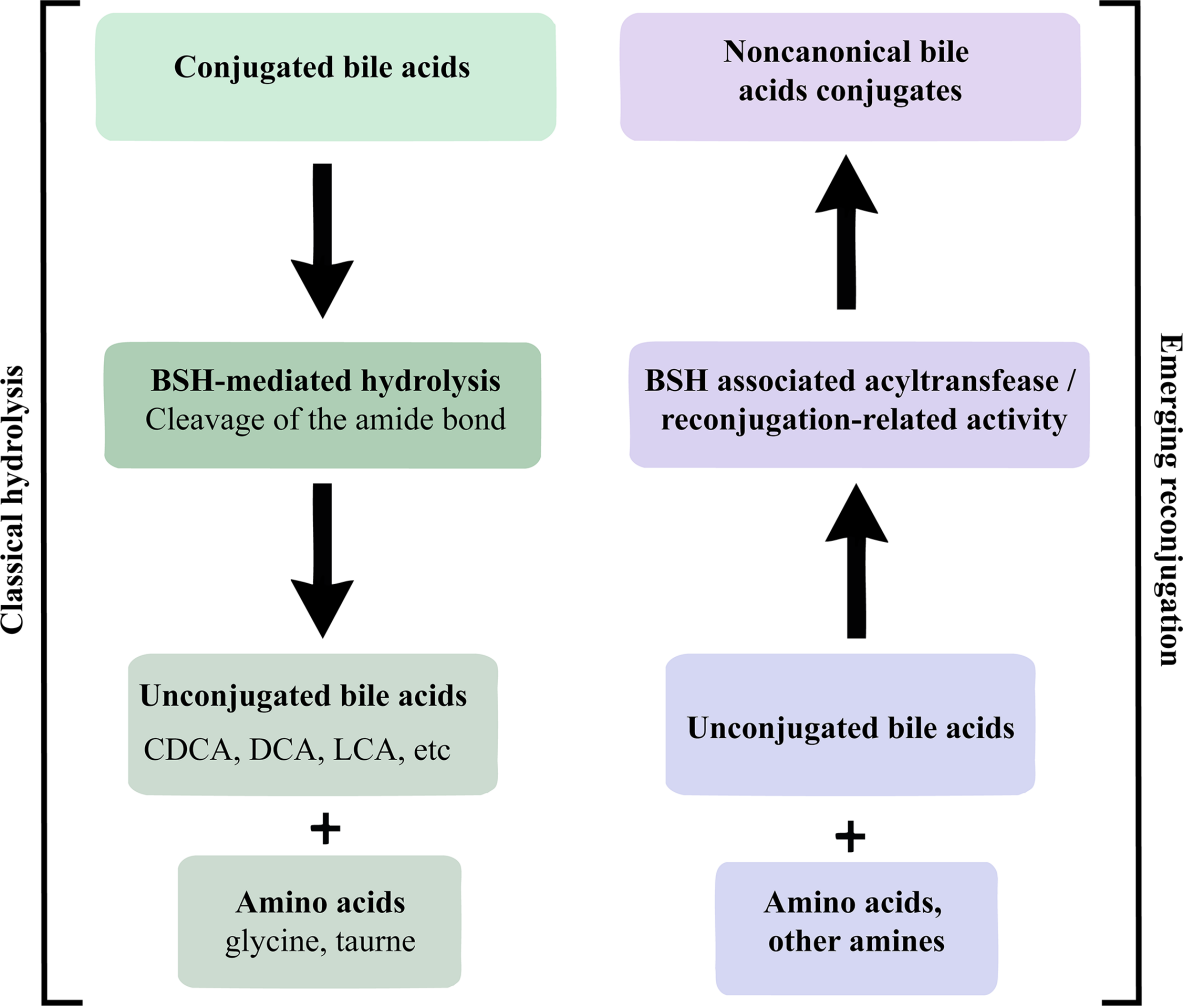
Classical deconjugation and emerging reconjugation-related activities of microbial BSH. BSH classically hydrolyzes the amide bond of glycine- or taurine-conjugated bile acids, generating unconjugated bile acids and releasing glycine or taurine. This reaction increases unconjugated bile acids availability and provides substrates for downstream microbial transformations. In addition to this canonical deconjugation reaction, emerging evidence indicates that certain BSH variants may participate in acyltransferase or reconjugation-related activity, leading to the formation of noncanonical bile acids conjugates from unconjugated bile acids and amino acids or amines.

Therefore, the importance of BSH catalysis extends beyond a single biochemical step. BSH-dependent deconjugation determines the initial substrate landscape for downstream bile acid transformations and receptor signaling. The classical deconjugation and emerging reconjugation-related activities of microbial BSH was shown in **Figure 1**.

## 3 Core mechanisms by which BSH regulates digestive homeostasis

### 3.1 Regulation of host metabolism through FXR and TGR5

Bile acid signaling is mediated primarily through the nuclear receptor FXR and the membrane receptor TGR5. FXR is widely regarded as a central regulator of bile acid homeostasis. In the intestine, FXR induces fibroblast growth factor 15/19 (FGF15/19) secretion, which suppresses hepatic bile acid synthesis through the gut–liver axis and modulates glucose and lipid metabolism (45, 46). In the liver, FXR further regulates lipid transport and glucose metabolic pathways through transcriptional networks (12, 28). TGR5 directly affects energy metabolism and endocrine regulation. The activation of TGR5 promotes incretin secretion, enhances energy expenditure and modulates immune cell function (47–50). Certain secondary bile acids, including LCA and DCA, serve as potent endogenous ligands of TGR5. The generation of these molecules depends on BSH-mediated deconjugation followed by downstream microbial metabolism (2, 50). Although TGR5 signaling confers metabolic benefits in several experimental models, systemic activation has also been associated with gallbladder filling and impaired emptying, highlighting the need to balance therapeutic efficacy and safety (47, 49). Moreover, the metabolic outcomes attributed to FXR or TGR5 signaling are not always consistent across studies (51–57). These discrepancies likely reflect differences in bile acid composition, the anatomical sites of receptor activation and disease stage rather than intrinsic properties of the receptor.

Within this receptor-dependent framework, the source and composition of bile acids emerge as key determinants of signaling output. By regulating the conversion between conjugated and unconjugated bile acids, BSH shapes the ligand spectrum available to FXR and TGR5 and influences their local exposure patterns (5, 22, 28). Individual bile acid species exhibit distinct agonistic or antagonistic effects on these receptors (2). The enrichment of specific bile acids in defined intestinal regions or their entry into the portal circulation therefore directly affects the magnitude and direction of receptor signaling. In addition to altering total bile acid levels, BSH exerts a more profound effect by reshaping bile acid signal quality, which is determined by the relative proportion and combination of specific bile acid subtypes (2, 28).

### 3.2 Maintenance of intestinal barrier integrity and immune regulation

In addition to metabolic regulation, BSH-mediated deconjugation contributes to intestinal barrier homeostasis and the shaping of the mucosal immune microenvironment. By modifying the physicochemical properties, spatial distribution and receptor activity of bile acid metabolites, BSH-dependent bile acid remodeling indirectly influences epithelial integrity, innate immune responses and adaptive immune balance, thereby affecting host responses to microbial stimulation (2, 58, 59).

Bile acids act as signaling molecules in epithelial cells. Through FXR and TGR5, they regulate tight junction protein expression, mucus layer integrity and epithelial renewal (2, 58). Under inflammatory conditions, FXR activation has been shown to preserve epithelial homeostasis and reduce tissue injury (58, 60). More broadly, bile acid remodeling can influence intestinal barrier function and inflammatory responses by altering the availability of FXR-, TGR5- and inflammasome-related bile acid signaling inputs (2, 59, 61, 62).

In the innate immune system, bile acids regulate inflammasome activation and innate immune cell function through receptor-mediated signaling. Changes in BSH activity may alter the generation and intestinal distribution of unconjugated and secondary bile acids that modulate inflammatory thresholds in macrophages and dendritic cells via FXR or TGR5 or related pathways (2, 59). Certain bile acid species inhibit NOD-like receptor family pyrin domain-containing 3 (NLRP3) inflammasome assembly and reduce proinflammatory cytokine release, thereby limiting excessive inflammation (59). Bile acid signaling also shapes adaptive immunity. Microbiota-derived bile acid metabolites regulate the balance between Tregs and Th17 cells (13), and further studies have indicated that these metabolites form an essential communication axis between the microbiota and the host immune system (63–65). Because BSH operates upstream in these metabolic pathways, fluctuations in its activity may alter the availability of immunoregulatory bile acids and thereby influence mucosal tolerance and inflammatory susceptibility.

Taken together, BSH-dependent bile acid remodeling links microbial metabolism to mucosal and systemic immune regulation through metabolite-mediated pathways. At the epithelial level, altered bile acid receptor signaling affects tight junction integrity, mucus barrier function, epithelial renewal and antimicrobial defense. In innate immunity, specific bile acid metabolites regulate macrophage and dendritic cell inflammatory thresholds through FXR/TGR5-related signaling and inflammasome-associated pathways. In adaptive immunity, microbiota-derived bile acid metabolites, including LCA-derived molecules, contribute to Treg–Th17 balance and mucosal tolerance. These immune-regulatory effects may also extend to cancer-related contexts, where BSH-dependent bile acid remodeling can influence chemokine production, immune-cell recruitment and tumor immune microenvironmental remodeling, as discussed in the following CRC section. Thus, BSH should be viewed as an upstream microbial metabolic enzyme that indirectly affects immune homeostasis through bile acid metabolite–receptor networks.

### 3.3 Influence on gut microbial composition and ecological balance

Bile acids exert antimicrobial activity and represent a major selective pressure that shapes gut microbial communities (66). Many bacteria express BSH to reduce the toxicity of conjugated bile acids, thereby increasing their survival within the intestinal environment. This bile acid–mediated selection favors microbes that can modify the local bile acid chemical landscape and confers ecological advantages during community succession. Changes in bile acid composition can selectively favor bile-tolerant or BSH-expressing taxa, including members of Firmicutes in some contexts, while suppressing bile acid–sensitive taxa (20, 66–68). By altering the local bile acid composition, BSH-expressing bacteria can create competitive advantages for themselves or coexisting species, thereby shaping the microbial community structure. This process can be conceptually viewed as niche engineering, whereby BSH-expressing bacteria reshape the local bile acid environment and alter the competitive dynamics of the microbiota (7, 65, 68). Experimental evidence further indicates that BSH activity directly influences bacterial gene expression and competitive fitness (67). Such “microbiota–bile acid–microbiota” feedback loops contribute to the dynamic stability of the intestinal ecosystem. For example, substrate specificity among distinct BSH variants in *Lactobacillus* determines bile tolerance and colonization capacity. Differential bile acid preferences influence bile acid–mediated growth inhibition and ultimately shape competitive outcomes among BSH-encoding strains in the gut environment (20).

Dietary patterns represent important external determinants of BSH abundance and the structure of BSH-active microbial communities (69). A ketogenic diet alters the composition of BSH-producing microbes and alters the overall functional capacity of intestinal BSH (4, 28). Increased intake of whole foods and dietary fiber is associated with elevated levels of glycoside hydrolases (GHs) and BSH, accompanied by a reduction in fecal conjugated bile acids (70, 71). These findings suggest that dietary components may regulate bile acid deconjugation through a GH–BSH functional axis and thereby influence microbial structure and metabolic health. Diet-induced alterations in BSH activity can further reshape the bile acid pool and affect host metabolic and immune homeostasis (72). Collectively, the interactions between microbial functional capacity and dietary inputs represent a key mechanism maintaining intestinal ecological balance.

**Figure 2:**
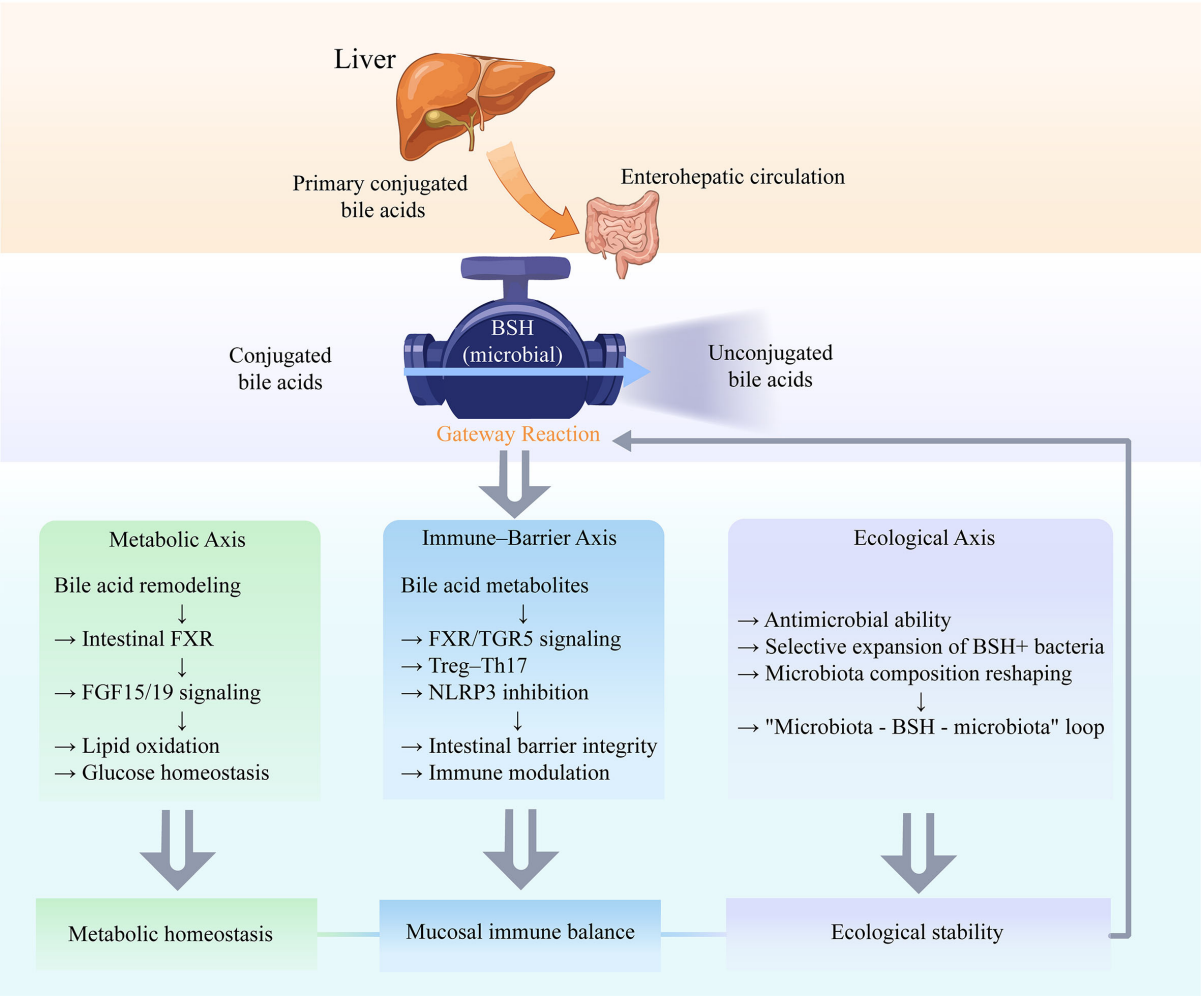
BSH as an upstream metabolic gatekeeper linking microbial bile acid metabolism to host physiological regulation. BSH, widely encoded by gut microbiota, catalyzes the deconjugation of conjugated bile acids and represents the gateway reaction of microbial bile acid transformation. By controlling the conversion of conjugated to unconjugated bile acids, BSH reshapes the composition and signaling properties of the intestinal bile acid pool. Changes in bile acid composition influence host receptor signaling and affect three interconnected domains: metabolic regulation (FXR–FGF15/19 signaling and hepatic metabolism), immune–barrier homeostasis (Treg–Th17 balance, inflammasome activity, and epithelial integrity), and microbial ecology through bile acid–microbiota feedback interactions.

The role of BSH as an upstream metabolic gatekeeper linking microbial bile acid metabolism to host physiological regulation is illustrated in **Figure 2**.

## 4 Associations between BSH dysregulation and major digestive diseases

### 4.1 MASLD

Accumulating evidence indicates that alterations in gut microbiota–derived BSH activity are closely linked to metabolic reprogramming during the development and progression of MASLD (73–75). Patients with MASLD exhibit systemic changes in bile acid composition and conjugation status (76), which correlate with the degree of insulin resistance, hepatic lipid accumulation and inflammatory activity.

Experimental and interventional studies suggest that BSH-associated pathways influence MASLD through modulation of gut–liver feedback signaling, particularly the intestinal FXR–FGF15/19 axis, thereby affecting hepatic cholesterol catabolism, fatty acid oxidation and glucose homeostasis (77, 78). After vertical sleeve gastrectomy, microbiota remodeling is accompanied by increased BSH activity and a greater proportion of unconjugated bile acids. These changes coincide with a reduced hepatic lipid burden and improved metabolic parameters (77, 79). Mechanistic studies further indicate that shifts in bile acid profiles modulate not only receptor signaling intensity but also inflammatory responses, oxidative stress and mitochondrial function, thereby influencing hepatocellular energy utilization and contributing to fibrosis progression (42, 80–82). In this context, BSH functions as an upstream integrator of multilayered metabolic regulation rather than as a regulator of a single pathway.

Importantly, the metabolic effects associated with BSH are not linearly beneficial. Excessive BSH activity may promote the overproduction of hydrophobic secondary bile acids (42, 80–82), which can exacerbate hepatocellular stress, impair mitochondrial function and accelerate the transition to steatohepatitis. These observations suggest that MASLD may require restoration of a balanced bile acid conversion network rather than nonspecific enhancement or inhibition of total BSH activity (83).

### 4.2 IBD

The pathogenesis of IBD is closely linked to impaired epithelial barrier function and disruption of immune homeostasis. As discussed in Section 3.2, bile acid metabolites contribute to epithelial integrity, innate immune regulation and Treg–Th17 balance through receptor-dependent and metabolite-specific pathways, including FXR/TGR5 signaling, inflammasome regulation and microbiota-derived secondary bile acid metabolites (2, 58). Changes in BSH-mediated bile acid deconjugation may therefore influence IBD progression indirectly by reshaping the bile acid metabolite pool and the corresponding receptor-signaling inputs.

Clinically, patients with IBD exhibit disturbed bile acid profiles, often characterized by an increased proportion of conjugated bile acids (11, 12, 61, 62). These alterations are associated with enhanced mucosal permeability, tight junction disruption and heightened inflammatory activity. Reduced or functionally imbalanced BSH activity may increase the proportion of conjugated bile acids and limit the availability of unconjugated bile acids and downstream secondary bile acid metabolites, thereby weakening bile acid receptor–dependent epithelial repair and anti-inflammatory signaling, including FXR/TGR5-related and inflammasome-associated pathways (58–62). Bile acid metabolites regulate inflammasome activation and macrophage inflammatory thresholds (59), and microbiota-derived bile acid derivatives have been shown to modulate Treg and Th17 differentiation (13). Impaired BSH-dependent substrate supply may reduce the availability of immunoregulatory bile acid metabolites. However, recent evidence further suggests that disease outcomes may depend on the balance between bile acid conjugation and hydrolysis rather than on the absolute level of BSH activity alone (12). Therefore, IBD-related BSH dysfunction should be interpreted as a disruption of bile acid conjugation–deconjugation balance rather than a simple decrease in total BSH activity. Such shifts in bile acid–dependent signaling align with the relapsing and chronic inflammatory nature of IBD.

Clinical and interventional studies provide support for this framework. Multiomics analyses have revealed a reduced abundance of BSH-producing microbes in IBD, accompanied by the depletion of anti-inflammatory secondary bile acids (12, 84). Consistently, regulatory T-cell–associated pathways are suppressed during active disease, whereas the restoration of bile acid metabolic capacity partially reverses immune imbalance (13, 85). In parallel, bile acid receptor signaling directly contributes to tight junction maintenance and epithelial repair (61, 62). Functional studies have further demonstrated that re-establishing BSH-associated metabolic axes or restoring bile acid conversion balance can attenuate mucosal inflammation and improve barrier integrity (86). Thus, therapeutic restoration of BSH-related bile acid metabolism in IBD should aim to recover a disease-appropriate bile acid profile rather than globally increasing BSH activity.

### 4.3 CRC

CRC is widely recognized as an inflammation-associated malignancy that is strongly shaped by its immune microenvironment. During CRC initiation and progression, BSH-mediated bile acid transformation influences tumor-associated ecological conditions. Alterations in bile acid composition affect not only tumor cell behavior but also immune cell recruitment and functional polarization, thereby modulating tumor growth and metastatic potential.

In patients with colorectal cancer liver metastasis, BSH-active microbial populations are reduced, accompanied by elevated levels of circulating conjugated bile acids (14). This shift promotes chemokine expression and enhances neutrophil recruitment to the liver, establishing a proinflammatory niche that supports metastatic colonization (14). The restoration of unconjugated bile acid proportions attenuates this prometastatic effect.

Beyond metastatic progression, aberrant BSH activity may also contribute to local tumor development through the modulation of immunosuppressive networks. Enrichment of high-BSH microbiota can increase the accumulation of specific secondary bile acids, which are associated with the expansion of immunosuppressive cell populations, including regulatory T cells (85, 87). This shift may dampen antitumor immunity and facilitate sustained tumor growth.

Therefore, in CRC, both insufficient and excessive BSH-associated bile acid remodeling may generate tumor-promoting immune ecological states, depending on tumor stage, anatomical site and bile acid profile.

An integrated overview of BSH in major digestive diseases is provided in **Table 1**.

**Table 1:** Integrated summary of BSH in digestive diseases.

Disease	Direction of BSH imbalance	Bile acid profile alteration	Dominant signaling axis	Key pathogenic mechanisms	Clinical implication
MASLD	Bidirectional (74, 77, 104)	Altered conjugated/unconjugated bile acid ratio Excessive hydrophobic secondary bile acids generation when overactivated (42, 74, 76, 80–82)	Intestinal FXR–FGF15/19–liver axis (77–79, 119)	Hepatic metabolic reprogramming Altered lipid oxidation Mitochondrial dysfunction Oxidative stress Inflammation amplification (42, 77–82)	Maintaining dynamic bile acid conversion balance may be more critical than simple up- or down-regulation of BSH (74, 83)
IBD	Predominantly decreased or functionally imbalanced (11, 12, 84)	Increased conjugated bile acids proportion Reduced anti-inflammatory secondary bile acids (11, 12, 84)	FXR/TGR5 immune–barrier axis (58, 59, 61)	Reduced Treg differentiation Enhanced Th17 polarization NLRP3 inflammasome activation Impaired tight junction integrity (13, 59, 61, 62)	Restoration of BSH–bile acid metabolic axis may improve mucosal immune balance and barrier function (84, 86)
CRC	Bidirectional (14, 87)	Elevated conjugated bile acids in metastatic context Secondary bile acids accumulation in high-BSH microbiota (14, 87)	Tumor immune microenvironment modulation (14, 15)	Neutrophil recruitment Expansion of immunosuppressive cell populations Inflammatory niche formation (14, 85, 87)	BSH acts as a microenvironmental switch and may require precision modulation depending on tumor stage (14, 85, 87)

BSH, bile salt hydrolase; MASLD, metabolic dysfunction–associated steatotic liver disease; IBD, inflammatory bowel disease; CRC, colorectal cancer.

## 5 Therapeutic strategies targeting BSH and future perspectives

### 5.1 Probiotic and prebiotic interventions

The identification and development of probiotic strains with defined and efficient BSH activity are one of the most direct strategies to modulate bile acid metabolism. Distinct bacterial strains encode BSH variants with marked differences in substrate specificity, which influence their ability to reshape bile acid pool composition and functional diversity (20, 28, 88, 89). In metabolic and hypercholesterolemia models, supplementation with BSH-active strains is frequently accompanied by improvements in lipid profiles and alterations in bile acid composition (90–92). However, the extent to which BSH activity directly drives observed host phenotypes remains to be fully established.

Compared with exogenous strain supplementation, prebiotic approaches indirectly modulate BSH functional capacity by altering microbial competitive dynamics (93–95). Dietary fiber has been shown to influence BSH activity through the restructuring of gut microbial communities (70, 96). Nevertheless, prebiotic-induced changes in BSH often occur alongside broader remodeling of microbial metabolic networks, making it difficult to isolate the independent contribution of BSH to therapeutic outcomes.

Advances in synthetic biology have introduced the possibility of precise BSH modulation (97, 98). Engineered probiotics can be designed to express defined BSH variants in response to specific environmental cues, such as inflammation, nutrient availability or circadian signals (99, 100), enabling controlled adjustment of bile acid metabolism. In parallel, activity-based chemical proteomic approaches allow the real-time profiling of BSH catalytic activity within complex microbial ecosystems (18, 101). The recognition of circadian oscillations in microbial functions (102, 103) further supports the concept of time-responsive engineered interventions. Despite these advances, clinical translation requires clearer mechanistic validation, strain-level specificity and predictive frameworks to ensure safety and reproducibility.

### 5.2 Small-molecule inhibitors and activators

Elevated BSH activity has been observed in subsets of patients with MASLD or liver fibrosis and is associated with altered bile acid profiles and disease severity (19, 104). Chemical probes and covalent inhibitors have been developed to suppress BSH catalytic activity without substantially impairing bacterial growth (32, 104, 105). By limiting bile acid deconjugation, these agents promote the retention of conjugated bile acids within the intestinal lumen and reduce downstream secondary bile acid formation, thereby altering the ligand landscape presented to host receptor networks. In animal models, such directional remodeling of bile acid composition is accompanied by improvements in metabolic or inflammatory phenotypes, supporting the feasibility of pharmacological targeting of BSH (105). Building on this strategy, AAA-10 has been developed as a gut-restricted pan-BSH inhibitor. This slow-acting compound with modifies the catalytic cysteine residue of BSH by irreversible covalent binding and achieves sustained enzymatic suppression while largely preserving overall microbial growth (106).

In contrast, in conditions characterized by reduced secondary bile acids and impaired immune regulation, such as IBD, the restoration or selective enhancement of specific BSH functions may help re-establish an immune-tolerant metabolic environment (13, 84, 107). However, compared with inhibition strategies, the pharmacological activation of BSH remains largely at the proof-of-concept stage. Existing approaches to augment BSH activity rely predominantly on supplementation with high-expressing strains or on the indirect modulation of microbial communities (108–110).

Given that BSH occupies an upstream position within the bile acid metabolic network, its modulation may be amplified across multiple regulatory layers. Microbiota-derived bile acid alterations can influence immune cell function through receptors such as FXR (111, 112) and drive cross-organ metabolic reprogramming along the gut–liver axis (113). Thus, even localized enzymatic intervention may have systemic consequences. Future small-molecule design will therefore require not only improved selectivity but also careful consideration of isoform-level functional redundancy and potential ecological compensation within the microbial community.

### 5.3 Dietary and lifestyle interventions

Dietary composition and intake patterns influence bile acid secretion and gut microbial structure, thereby indirectly modulating BSH activity and bile acid pool composition (114, 115). Dietary patterns are closely associated with the abundance of BSH-producing microbes and with the overall BSH functional capacity in the gut (4, 70, 116, 117). In metabolic and inflammatory contexts, specific dietary regimens can rapidly reshape microbial communities, accompanied by changes in BSH activity and bile acid composition, which in turn affect the FXR–FGF15/19 axis and downstream metabolic feedback loops (117–121). By altering the bile acid ligands that enter host receptor networks, dietary interventions may generate amplified downstream effects. However, much of the mechanistic evidence derives from animal models, and the extent to which these findings predict outcomes in human disease remains to be fully established.

In human cohorts, fecal microbiota transplantation (FMT) alters the intestinal bile acid composition and is associated with improvements in host metabolic parameters (122, 123). Although FMT is not BSH specific, these findings support the broader concept that restructuring microbial bile acid transformation capacity can reprogram host signaling environments with therapeutic potential.

**Figure 3:**
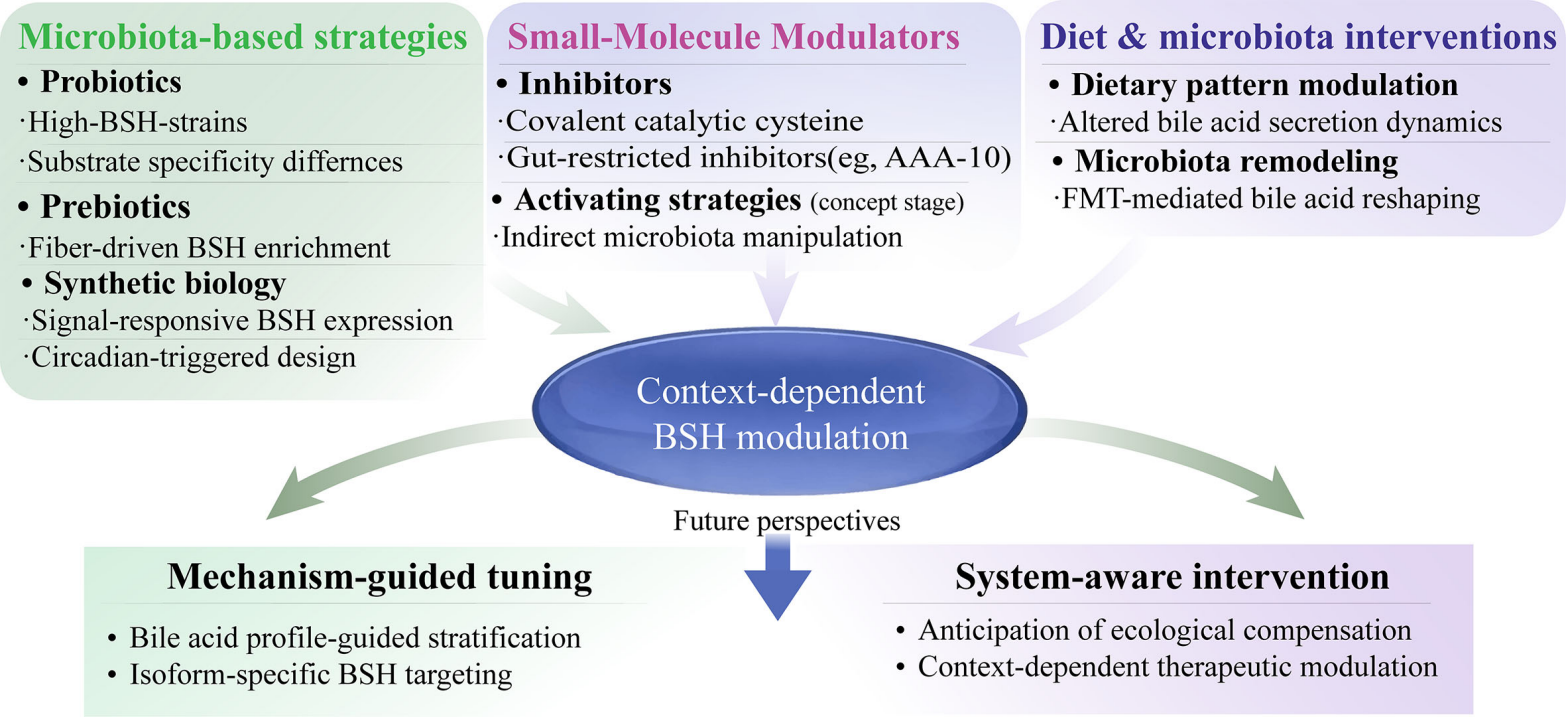
Therapeutic strategies targeting BSH and future perspectives. Multiple therapeutic approaches have been proposed to modulate BSH activity and reshape bile acid metabolism. Microbiota-based strategies, including probiotics, prebiotics and synthetic biology approaches, aim to modify microbial BSH capacity and substrate specificity. Small-molecule modulators directly regulate BSH catalytic activity, whereas dietary and microbiota-remodeling interventions indirectly influence bile acid transformation through changes in microbial community structure. These strategies converge on context-dependent BSH modulation, enabling precision tuning of bile acid metabolism. Future approaches may incorporate bile acid profile–guided stratification and isoform-specific BSH targeting, together with system-aware interventions that anticipate ecological compensation and enable context-dependent therapeutic modulation.

**Figure 3** summarizes therapeutic strategies targeting BSH and outlines future perspectives.

## 6 Conclusions and future perspectives

BSH occupies a strategic upstream position within the microbial bile acid transformation network and functions less as a simple hydrolytic enzyme than as a metabolic gatekeeper that shapes the quality, distribution, and signaling potential of the intestinal bile acid pool. By redistributing bile acid species, BSH determines the ligand landscape available to FXR, TGR5 and related pathways. Through this mechanism, BSH integrates microbial ecology with host metabolic, immune, and barrier regulation across the gut–liver axis.

Importantly, the biological impact of BSH is disease- and context-specific rather than uniformly protective or deleterious. This context dependency argues against global activation or inhibition of BSH as a universal therapeutic strategy and instead supports the restoration of disease-appropriate bile acid signaling. Therapeutic strategies targeting BSH, including strain-specific probiotics, small-molecule modulators, and ecological interventions, hold translational promise but must be guided by a precise framework. Future efforts should integrate isoform-resolved BSH mapping, bile acid metabolomic signatures, host immune context, and ecological compensation to guide precision microbial interventions. This strategy may enable the development of bile acid–informed biomarkers and context-aware microbial therapeutics, facilitating the translation of mechanistic insights into clinically actionable interventions for digestive diseases.

## References

[1] Molinaro A Wahlström A Marschall HU . Role of bile acids in metabolic control. Trends Endocrinol Metab. (2018) 29:31–41. doi: 10.1016/j.tem.2017.11.002 29195686

[2] Fleishman JS Kumar S . Bile acid metabolism and signaling in health and disease: molecular mechanisms and therapeutic targets. Signal Transduction Targeted Ther. (2024) 9:97. doi: 10.1038/s41392-024-01811-6 38664391 PMC11045871

[3] Bourgin M Kriaa A Mkaouar H Mariaule V Jablaoui A Maguin E . Bile salt hydrolases: At the crossroads of microbiota and human health. Microorganisms. (2021) 9:1122. doi: 10.3390/microorganisms9061122 34067328 PMC8224655

[4] Jia B Park D Chun BH Hahn Y Jeon CO . Diet-related alterations of gut bile salt hydrolases determined using a metagenomic analysis of the human microbiome. Int J Mol Sci. (2021) 22:3652. doi: 10.3390/ijms22073652 33915727 PMC8038126

[5] Rimal B Collins SL Tanes CE Rocha ER Granda MA Solanki S . Bile salt hydrolase catalyses formation of amine-conjugated bile acids. Nature. (2024) 626:859–63. doi: 10.1038/s41586-023-06990-w 38326609 PMC10881385

[6] Funabashi M Grove TL Wang M Varma Y McFadden ME Brown LC . A metabolic pathway for bile acid dehydroxylation by the gut microbiome. Nature. (2020) 582:566–70. doi: 10.1038/s41586-020-2396-4 32555455 PMC7319900

[7] Guzior DV Quinn RA . Review: microbial transformations of human bile acids. Microbiome. (2021) 9:140. doi: 10.1186/s40168-021-01101-1 34127070 PMC8204491

[8] Shu JZ Huang YH He XH Liu FY Liang QQ Yong XT . Gut microbiota differences, metabolite changes, and disease intervention during metabolic - dysfunction - related fatty liver progression. World J Hepatol. (2025) 17:103854. doi: 10.4254/wjh.v17.i3.103854 40177201 PMC11959672

[9] Ni Y Lu M Xu Y Wang Q Gu X Li Y . The role of gut microbiota-bile acids axis in the progression of non-alcoholic fatty liver disease. Front Microbiol. (2022) 13:908011. doi: 10.3389/fmicb.2022.908011 35832821 PMC9271914

[10] Zeng F Su X Liang X Liao M Zhong H Xu J . Gut microbiome features and metabolites in non-alcoholic fatty liver disease among community-dwelling middle-aged and older adults. BMC Med. (2024) 22:104. doi: 10.1186/s12916-024-03317-y 38454425 PMC10921631

[11] Heinken A Ravcheev DA Baldini F Heirendt L Fleming RMT Thiele I . Systematic assessment of secondary bile acid metabolism in gut microbes reveals distinct metabolic capabilities in inflammatory bowel disease. Microbiome. (2019) 7:75. doi: 10.1186/s40168-019-0689-3 31092280 PMC6521386

[12] Fu Y Guzior DV Okros M Bridges C Rosset SL González CT . Balance between bile acid conjugation and hydrolysis activity can alter outcomes of gut inflammation. Nat Commun. (2025) 16:3434. doi: 10.1038/s41467-025-58649-x 40210868 PMC11985902

[13] Hang S Paik D Yao L Kim E Trinath J Lu J . Bile acid metabolites control T(H)17 and T(reg) cell differentiation. Nature. (2019) 576:143–8. doi: 10.1038/s41586-019-1785-z 31776512 PMC6949019

[14] Zheng Z Yuan F Li J Zhu X Jia R Wei J . Gut microbiota-mediated bile acid metabolism regulates colorectal cancer liver metastasis by altering neutrophil recruitment. Cancer Res. (2025) 85:4081–98. doi: 10.1158/0008-5472.Can-24-4425 40865051

[15] Li C Xing X Li M Liu Y Huang S Zhu T . Bile acids produced by gut microbiota activate TGR5 to promote colorectal liver metastasis progression by inducing MDSCs infiltration in liver. Int Immunopharmacol. (2025) 158:114829. doi: 10.1016/j.intimp.2025.114829 40367692

[16] Kombala CJ Agrawal N Sveistyte A Karatsoreos IN Van Dongen HPA Brandvold KR . Profiling rhythmicity of bile salt hydrolase activity in the gut lumen with a rapid fluorescence assay. Org Biomol Chem. (2023) 21:4028–38. doi: 10.1039/d2ob02257e 36810586 PMC10191106

[17] Xu F Guo F Hu XJ Lin J . Crystal structure of bile salt hydrolase from Lactobacillus salivarius. Acta Crystallogr F Struct Biol Commun. (2016) 72:376–81. doi: 10.1107/s2053230x16005707 27139829 PMC4854565

[18] Bracken AK Malarney KP Chang PV . Chemical proteomics reveals regulation of bile salt hydrolases via oxidative post-translational modifications. J Am Chem Soc. (2026) 148:5378–86. doi: 10.1021/jacs.5c18912 41612134 PMC12903841

[19] Joyce SA MacSharry J Casey PG Kinsella M Murphy EF Shanahan F . Regulation of host weight gain and lipid metabolism by bacterial bile acid modification in the gut. PNAS. (2014) 111:7421–6. doi: 10.1073/pnas.1323599111 24799697 PMC4034235

[20] Foley MH O'Flaherty S Allen G Rivera AJ Stewart AK Barrangou R . Lactobacillus bile salt hydrolase substrate specificity governs bacterial fitness and host colonization. PNAS. (2021) 118:e2017709118. doi: 10.1073/pnas.2017709118 33526676 PMC8017965

[21] Ruiz L Sánchez B Margolles A . Determination of bile salt hydrolase activity in bifidobacteria. Methods Mol Biol. (2021) 2278:149–55. doi: 10.1007/978-1-0716-1274-3_13 33649955

[22] Guzior DV Okros M Shivel M Armwald B Bridges C Fu Y . Bile salt hydrolase acyltransferase activity expands bile acid diversity. Nature. (2024) 626:852–8. doi: 10.1038/s41586-024-07017-8 38326608

[23] Kvitne KE Allaband C Onuora JC Perry D Zuffa S Patel L . Environmental and maternal imprints on infant gut metabolic development. Cell Host Microbe. (2025) 33:2130–2147.e7. doi: 10.1016/j.chom.2025.11.002 41308639 PMC12875530

[24] Karlov DS Long SL Zeng X Xu F Lal K Cao L . Characterization of the mechanism of bile salt hydrolase substrate specificity by experimental and computational analyses. Structure. (2023) 31:629–638.e5. doi: 10.1016/j.str.2023.02.014 36963397

[25] Dong Z Lee BH . Bile salt hydrolases: Structure and function, substrate preference, and inhibitor development. Protein Sci. (2018) 27:1742–54. doi: 10.1002/pro.3484 30098054 PMC6199152

[26] Fang F Li Y Bumann M Raftis EJ Casey PG Cooney JC . Allelic variation of bile salt hydrolase genes in Lactobacillus salivarius does not determine bile resistance levels. J Bacteriol. (2009) 191:5743–57. doi: 10.1128/jb.00506-09 19592587 PMC2737978

[27] Kumar R Grover S Batish VK . Bile salt hydrolase (Bsh) activity screening of lactobacilli: *In vitro* selection of indigenous Lactobacillus strains with potential bile salt hydrolysing and cholesterol-lowering ability. Probiotics Antimicrob Proteins. (2012) 4:162–72. doi: 10.1007/s12602-012-9101-3 26782042

[28] Foley MH Walker ME Stewart AK O'Flaherty S Gentry EC Patel S . Bile salt hydrolases shape the bile acid landscape and restrict Clostridioides difficile growth in the murine gut. Nat Microbiol. (2023) 8:611–28. doi: 10.1038/s41564-023-01337-7 36914755 PMC10066039

[29] Wang H Chen J Hollister K Sowers LC Forman BM . Endogenous bile acids are ligands for the nuclear receptor FXR/BAR. Mol Cell. (1999) 3:543–53. doi: 10.1016/s1097-2765(00)80348-2 10360171

[30] Duboc H Taché Y Hofmann AF . The bile acid TGR5 membrane receptor: from basic research to clinical application. Dig Liver Dis. (2014) 46:302–12. doi: 10.1016/j.dld.2013.10.021 24411485 PMC5953190

[31] Li W Hang S Fang Y Bae S Zhang Y Zhang M . A bacterial bile acid metabolite modulates T(reg) activity through the nuclear hormone receptor NR4A1. Cell Host Microbe. (2021) 29:1366–1377.e9. doi: 10.1016/j.chom.2021.07.013 34416161 PMC9064000

[32] Parasar B Zhou H Xiao X Shi Q Brito IL Chang PV . Chemoproteomic profiling of gut microbiota-associated bile salt hydrolase activity. ACS Cent Sci. (2019) 5:867–73. doi: 10.1021/acscentsci.9b00147 31139722 PMC6535767

[33] Han L Pendleton A Singh A Xu R Scott SA Palma JA . Chemoproteomic profiling of substrate specificity in gut microbiota-associated bile salt hydrolases. Cell Chem Biol. (2025) 32:145–156.e9. doi: 10.1016/j.chembiol.2024.05.009 38889717 PMC11632149

[34] Jones BV Begley M Hill C Gahan CGM Marchesi JR . Functional and comparative metagenomic analysis of bile salt hydrolase activity in the human gut microbiome. PNAS. (2008) 105:13580–5. doi: 10.1073/pnas.0804437105 18757757 PMC2533232

[35] Fallingborg J . Intraluminal pH of the human gastrointestinal tract. Dan Med Bull. (1999) 46:183–96. 10421978

[36] Begley M Hill C Gahan CGM . Bile salt hydrolase activity in probiotics. Appl Environ Microbiol. (2006) 72:1729–38. doi: 10.1128/AEM.72.3.1729-1738.2006 16517616 PMC1393245

[37] Kusada H Arita M Tohno M Tamaki H . Isolation of a highly thermostable bile salt hydrolase with broad substrate specificity from Lactobacillus paragasseri. Front Microbiol. (2022) 13:810872. doi: 10.3389/fmicb.2022.810872 35250928 PMC8893165

[38] Agolino G Pino A Vaccalluzzo A Cristofolini M Solieri L Caggia C . Bile salt hydrolase: The complexity behind its mechanism in relation to lowering-cholesterol lactobacilli probiotics. J Funct Foods. (2024) 120:106357. doi: 10.1016/j.jff.2024.106357 38826717

[39] Wise JL Cummings BP . The 7-α-dehydroxylation pathway: An integral component of gut bacterial bile acid metabolism and potential therapeutic target. Front Microbiol. (2022) 13:1093420. doi: 10.3389/fmicb.2022.1093420 36699589 PMC9868651

[40] Francini E Badillo Pazmay GV Fumarola S Procopio AD Olivieri F Marchegiani F . Bi-directional relationship between bile acids (BAs) and gut microbiota (GM): UDCA/TUDCA, probiotics, and dietary interventions in elderly people. Int J Mol Sci. (2025) 26:1759. doi: 10.3390/ijms26041759 40004221 PMC11855466

[41] Kriaa A Bourgin M Potiron A Mkaouar H Jablaoui A Gérard P . Microbial impact on cholesterol and bile acid metabolism: current status and future prospects. J Lipid Res. (2019) 60:323–32. doi: 10.1194/jlr.R088989 30487175 PMC6358303

[42] Li Z Yuan H Chu H Yang L . The crosstalk between gut microbiota and bile acids promotes the development of non-alcoholic fatty liver disease. Microorganisms. (2023) 11:2059. doi: 10.3390/microorganisms11082059 37630619 PMC10459427

[43] Lynch LE Hair AB Soni KG Yang H Gollins LA Narvaez-Rivas M . Cholestasis impairs gut microbiota development and bile salt hydrolase activity in preterm neonates. Gut Microbes. (2023) 15:2183690. doi: 10.1080/19490976.2023.2183690 36843227 PMC9980517

[44] Li M Liu S Wang M Hu H Yin J Liu C . Gut microbiota dysbiosis associated with bile acid metabolism in neonatal cholestasis disease. Sci Rep. (2020) 10:7686. doi: 10.1038/s41598-020-64728-4 32377002 PMC7203226

[45] Qiu Y Yu J Ji X Yu H Xue M Zhang F . Ileal FXR-FGF15/19 signaling activation improves skeletal muscle loss in aged mice. Mech Ageing Dev. (2022) 202:111630. doi: 10.1016/j.mad.2022.111630 35026209

[46] Sun L Xie C Wang G Wu Y Wu Q Wang X . Gut microbiota and intestinal FXR mediate the clinical benefits of metformin. Nat Med. (2018) 24:1919–29. doi: 10.1038/s41591-018-0222-4 30397356 PMC6479226

[47] Wang Q Lin H Shen C Zhang M Wang X Yuan M . Gut microbiota regulates postprandial GLP-1 response via ileal bile acid-TGR5 signaling. Gut Microbes. (2023) 15:2274124. doi: 10.1080/19490976.2023.2274124 37942583 PMC10730136

[48] Zangerolamo L Carvalho M Barbosa HCL . The critical role of the bile acid receptor TGR5 in energy homeostasis: Insights into physiology and therapeutic potential. Int J Mol Sci. (2025) 26:6547. doi: 10.3390/ijms26146547 40724796 PMC12294878

[49] van Nierop FS Scheltema MJ Eggink HM Pols TW Sonne DP Knop FK . Clinical relevance of the bile acid receptor TGR5 in metabolism. Lancet Diabetes Endocrinol. (2017) 5:224–33. doi: 10.1016/S2213-8587(16)30155-3 27639537

[50] Watanabe M Houten SM Mataki C Christoffolete MA Kim BW Sato H . Bile acids induce energy expenditure by promoting intracellular thyroid hormone activation. Nature. (2006) 439:484–9. doi: 10.1038/nature04330 16400329

[51] Jiang C Xie C Li F Zhang L Nichols RG Krausz KW . Intestinal farnesoid X receptor signaling promotes nonalcoholic fatty liver disease. J Clin Invest. (2014) 125:386–402. doi: 10.1172/JCI76738 25500885 PMC4382255

[52] Ma K Saha PK Chan L Moore DD . Farnesoid X receptor is essential for normal glucose homeostasis. J Clin Invest. (2006) 116:1102–9. doi: 10.1172/jci25604 16557297 PMC1409738

[53] Adorini L Trauner M . FXR agonists in NASH treatment. J Hepatol. (2023) 79:1317–31. doi: 10.1016/j.jhep.2023.07.034 37562746

[54] Beau A Benoit B Le Barz M Meugnier E Penhoat A Calzada C . Inhibition of intestinal FXR activity as a possible mechanism for the beneficial effects of a probiotic mix supplementation on lipid metabolism alterations and weight gain in mice fed a high fat diet. Gut Microbes. (2023) 15:2281015. doi: 10.1080/19490976.2023.2281015 37985749 PMC10730200

[55] Thomas C Gioiello A Noriega L Strehle A Oury J Rizzo G . TGR5-mediated bile acid sensing controls glucose homeostasis. Cell Metab. (2009) 10:167–77. doi: 10.1016/j.cmet.2009.08.001 19723493 PMC2739652

[56] Vassileva G Hu W Hoos L Tetzloff G Yang S Liu L . Gender-dependent effect of Gpbar1 genetic deletion on the metabolic profiles of diet-induced obese mice. J Endocrinol. (2010) 205:225–32. doi: 10.1677/joe-10-0009 20354075

[57] Bhimanwar RS Mittal A Chaudhari S Sharma V . Recent advancements in the structural exploration of TGR5 agonists for diabetes treatment. RSC Med Chem. (2024) 15:3026–37. doi: 10.1039/d4md00473f 39309359 PMC11411620

[58] Inagaki T Moschetta A Lee Y-K Peng L Zhao G Downes M . Regulation of antibacterial defense in the small intestine by the nuclear bile acid receptor. PNAS. (2006) 103:3920–5. doi: 10.1073/pnas.0509592103 16473946 PMC1450165

[59] Guo C Xie S Chi Z Zhang J Liu Y Zhang L . Bile acids control inflammation and metabolic disorder through inhibition of NLRP3 inflammasome. Immunity. (2016) 45:802–16. doi: 10.1016/j.immuni.2016.09.008 27692610

[60] Gadaleta RM van Erpecum KJ Oldenburg B Willemsen EC Renooij W Murzilli S . Farnesoid X receptor activation inhibits inflammation and preserves the intestinal barrier in inflammatory bowel disease. Gut. (2011) 60:463–72. doi: 10.1136/gut.2010.212159 21242261

[61] Biagioli M Marchianò S Carino A Di Giorgio C Santucci L Distrutti E . Bile acids activated receptors in inflammatory bowel disease. Cells. (2021) 10:1281. doi: 10.3390/cells10061281 34064187 PMC8224328

[62] Calzadilla N Comiskey SM Dudeja PK Saksena S Gill RK Alrefai WA . Bile acids as inflammatory mediators and modulators of intestinal permeability. Front Immunol. (2022) 13:1021924. doi: 10.3389/fimmu.2022.1021924 36569849 PMC9768584

[63] Paik D Yao L Zhang Y Bae S D'Agostino GD Zhang M . Human gut bacteria produce τ(η)17-modulating bile acid metabolites. Nature. (2022) 603:907–12. doi: 10.1038/s41586-022-04480-z 35296854 PMC9132548

[64] Campbell C McKenney PT Konstantinovsky D Isaeva OI Schizas M Verter J . Bacterial metabolism of bile acids promotes generation of peripheral regulatory T cells. Nature. (2020) 581:475–9. doi: 10.1038/s41586-020-2193-0 32461639 PMC7540721

[65] Lee MH Nuccio SP Mohanty I Hagey LR Dorrestein PC Chu H . How bile acids and the microbiota interact to shape host immunity. Nat Rev Immunol. (2024) 24:798–809. doi: 10.1038/s41577-024-01057-x 39009868

[66] Larabi AB Masson HLP Bäumler AJ . Bile acids as modulators of gut microbiota composition and function. Gut Microbes. (2023) 15:2172671. doi: 10.1080/19490976.2023.2172671 36740850 PMC9904317

[67] Núñez-Sánchez MA Herisson FM Keane JM García-González N Rossini V Pinhiero J . Microbial bile salt hydrolase activity influences gene expression profiles and gastrointestinal maturation in infant mice. Gut Microbes. (2022) 14:2149023. doi: 10.1080/19490976.2022.2149023 36420990 PMC9704388

[68] Ridlon JM Harris SC Bhowmik S Kang D-J Hylemon PB . Consequences of bile salt biotransformations by intestinal bacteria. Gut Microbes. (2016) 7:22–39. doi: 10.1080/19490976.2015.1127483 26939849 PMC4856454

[69] Devlin AS Fischbach MA . A biosynthetic pathway for a prominent class of microbiota-derived bile acids. Nat Chem Biol. (2015) 11:685–90. doi: 10.1038/nchembio.1864 26192599 PMC4543561

[70] Kastl A Zong W Gershuni VM Friedman ES Tanes C Boateng A . Dietary fiber-based regulation of bile salt hydrolase activity in the gut microbiota and its relevance to human disease. Gut Microbes. (2022) 14:2083417. doi: 10.1080/19490976.2022.2083417 35658830 PMC9176262

[71] Lin Z Ma X . Dietary nutrients mediate crosstalk between bile acids and gut microbes in animal host metabolism. Crit Rev Food Sci Nutr. (2023) 63:9315–29. doi: 10.1080/10408398.2022.2067118 35507502

[72] Wahlström A Sayin Sama I Marschall H-U Bäckhed F . Intestinal crosstalk between bile acids and microbiota and its impact on host metabolism. Cell Metab. (2016) 24:41–50. doi: 10.1016/j.cmet.2016.05.005 27320064

[73] Hamamah S Iatcu OC Covasa M . Dietary influences on gut microbiota and their role in metabolic dysfunction-associated steatotic liver disease (MASLD). Nutrients. (2025) 17:143. doi: 10.3390/nu17010143 39796579 PMC11722922

[74] Zhao W Wang H Zheng M Ni Y . Bile salt hydrolase: a key player in gut microbiota and its implications for metabolic dysfunction-associated steatotic liver disease. Microbiome Res Rep. (2025) 4:28. doi: 10.20517/mrr.2025.12 41133099 PMC12540048

[75] Schnabl B Damman CJ Carr RM . Metabolic dysfunction–associated steatotic liver disease and the gut microbiome: pathogenic insights and therapeutic innovations. J Clin Invest. (2025) 135:e186423. doi: 10.1172/JCI186423 40166938 PMC11957707

[76] Lyu S Yang J Xin X Sun Q Cai B Wang X . Characteristics of serum bile acid profiles among individuals with metabolic dysfunction-associated steatotic liver disease. BMC Gastroenterol. (2025) 25:334. doi: 10.1186/s12876-025-03903-1 40325371 PMC12054156

[77] Xia Y Yang J Lu S Cheng W Ren M Liu Z . Microbial changes resulting from VSG attenuate MASLD by modulating bile acid metabolism and the intestinal FXR-FGF19 axis. mSystems. (2025) 10:e0063425. doi: 10.1128/msystems.00634-25 41114583 PMC12625774

[78] Lau H-H Zhang X Yu J . Gut microbiome in metabolic dysfunction-associated steatotic liver disease and associated hepatocellular carcinoma. Nat Rev Gastroenterol Hepatol. (2025) 22:619–38. doi: 10.1038/s41575-025-01089-1 40624229

[79] Ryan KK Tremaroli V Clemmensen C Kovatcheva-Datchary P Myronovych A Karns R . FXR is a molecular target for the effects of vertical sleeve gastrectomy. Nature. (2014) 509:183–8. doi: 10.1038/nature13135 24670636 PMC4016120

[80] Jiao N Baker SS Chapa-Rodriguez A Liu W Nugent CA Tsompana M . Suppressed hepatic bile acid signalling despite elevated production of primary and secondary bile acids in NAFLD. Gut. (2018) 67:1881–91. doi: 10.1136/gutjnl-2017-314307 28774887

[81] Holtmann TM Inzaugarat ME Knorr J Geisler L Schulz M Bieghs V . Bile acids activate NLRP3 inflammasome, promoting murine liver inflammation or fibrosis in a cell type-specific manner. Cells. (2021) 10:2618. doi: 10.3390/cells10102618 34685598 PMC8534222

[82] Xie G Jiang R Wang X Liu P Zhao A Wu Y . Conjugated secondary 12α-hydroxylated bile acids promote liver fibrogenesis. eBioMedicine. (2021) 66:103290. doi: 10.1016/j.ebiom.2021.103290 33752128 PMC8010625

[83] Cheng Z Chen Y Schnabl B Chu H Yang L . Bile acid and nonalcoholic steatohepatitis: molecular insights and therapeutic targets. J Adv Res. (2024) 59:173–87. doi: 10.1016/j.jare.2023.06.009 37356804 PMC11081971

[84] Zhou C Wang Y Li C Xie Z Dai L . Amelioration of colitis by a gut bacterial consortium producing anti-inflammatory secondary bile acids. Microbiol Spectr. (2023) 11:e0333022. doi: 10.1128/spectrum.03330-22 36943054 PMC10101101

[85] Song X Sun X Oh SF Wu M Zhang Y Zheng W . Microbial bile acid metabolites modulate gut RORγ+ regulatory T cell homeostasis. Nature. (2020) 577:410–5. doi: 10.1038/s41586-019-1865-0 31875848 PMC7274525

[86] Yang Q Liu J Lyu S Li S Han Q Ma C . Ovalbumin peptides restore intestinal barrier integrity via gut-liver axis modulation of bile salt hydrolase and bile acids crosstalk. J Agric Food Chem. (2025) 73:24741–52. doi: 10.1021/acs.jafc.5c07236 40958146

[87] Sun L Zhang Y Cai J Rimal B Rocha ER Coleman JP . Bile salt hydrolase in non-enterotoxigenic Bacteroides potentiates colorectal cancer. Nat Commun. (2023) 14:755. doi: 10.1038/s41467-023-36089-9 36765047 PMC9918522

[88] Jiang J Hang X Zhang M Liu X Li D Yang H . Diversity of bile salt hydrolase activities in different lactobacilli toward human bile salts. Ann Microbiol. (2009) 60:81–8. doi: 10.1007/s13213-009-0004-9 30311153

[89] Chen Y Chaudhari SN Harris DA Roberts CF Moscalu A Mathur V . A small intestinal bile acid modulates the gut microbiome to improve host metabolic phenotypes following bariatric surgery. Cell Host Microbe. (2024) 32:1315–1330.e5. doi: 10.1016/j.chom.2024.06.014 39043190 PMC11332993

[90] Hernández-Gómez JG López-Bonilla A Trejo-Tapia G Ávila-Reyes SV Jiménez-Aparicio AR Hernández-Sánchez H . *In vitro* bile salt hydrolase (BSH) activity screening of different probiotic microorganisms. Foods. (2021) 10:674. doi: 10.3390/foods10030674 33810002 PMC8004636

[91] Jiang J Wu C Zhang C Zhao J Yu L Zhang H . Effects of probiotic supplementation on cardiovascular risk factors in hypercholesterolemia: a systematic review and meta-analysis of randomized clinical trial. J Funct Foods. (2020) 74:104177. doi: 10.1016/j.jff.2020.104177 38826717

[92] Liu Y Kuang W Li M Wang Z Liu Y Zhao M . Cholesterol-lowering mechanism of Lactobacillus bile salt hydrolase through regulation of Bifidobacterium pseudolongum in the gut microbiota. Nutrients. (2025) 17:3019. doi: 10.3390/nu17183019 41010544 PMC12472961

[93] Adebola OO Corcoran O Morgan WA . Prebiotics may alter bile salt hydrolase activity: possible implications for cholesterol metabolism. PharmaNutrition. (2020) 12:100182. doi: 10.1016/j.phanu.2020.100182 38826717

[94] Khodakivskyi PV Lauber CL Yevtodiyenko A Bazhin AA Bruce S Ringel-Kulka T . Noninvasive imaging and quantification of bile salt hydrolase activity: from bacteria to humans. Sci Adv. (2021) 7:eaaz9857. doi: 10.1126/sciadv.aaz9857 33536224 PMC7857686

[95] Deehan EC Yang C Perez-Muñoz ME Nguyen NK Cheng CC Triador L . Precision microbiome modulation with discrete dietary fiber structures directs short-chain fatty acid production. Cell Host Microbe. (2020) 27:389–404.e6. doi: 10.1016/j.chom.2020.01.006 32004499

[96] David LA Maurice CF Carmody RN Gootenberg DB Button JE Wolfe BE . Diet rapidly and reproducibly alters the human gut microbiome. Nature. (2014) 505:559–63. doi: 10.1038/nature12820 24336217 PMC3957428

[97] Isabella VM Ha BN Castillo MJ Lubkowicz DJ Rowe SE Millet YA . Development of a synthetic live bacterial therapeutic for the human metabolic disease phenylketonuria. Nat Biotechnol. (2018) 36:857–64. doi: 10.1038/nbt.4222 30102294

[98] Vockley J Sondheimer N Puurunen M Diaz GA Ginevic I Grange DK . Efficacy and safety of a synthetic biotic for treatment of phenylketonuria: a phase 2 clinical trial. Nat Metab. (2023) 5:1685–90. doi: 10.1038/s42255-023-00897-6 37770764

[99] Riglar DT Silver PA . Engineering bacteria for diagnostic and therapeutic applications. Nat Rev Microbiol. (2018) 16:214–25. doi: 10.1038/nrmicro.2017.172 29398705

[100] Meng J Liu S Wu X . Engineered probiotics as live biotherapeutics for diagnosis and treatment of human diseases. Crit Rev Microbiol. (2024) 50:300–14. doi: 10.1080/1040841x.2023.2190392 36946080

[101] Han L Xu R Conwell AN Takahashi S Parasar B Chang PV . Bile salt hydrolase activity-based probes for monitoring gut microbial bile acid metabolism. ChemBioChem. (2024) 25:e202300821. doi: 10.1002/cbic.202300821 38564329 PMC11102598

[102] Thaiss CA Zeevi D Levy M Zilberman-Schapira G Suez J Tengeler AC . Transkingdom control of microbiota diurnal oscillations promotes metabolic homeostasis. Cell. (2014) 159:514–29. doi: 10.1016/j.cell.2014.09.048 25417104

[103] Litichevskiy L Thaiss CA . The oscillating gut microbiome and its effects on host circadian biology. Annu Rev Nutr. (2022) 42:145–64. doi: 10.1146/annurev-nutr-062320-111321 35576592

[104] Jones EV Wang Y Wei W Reed JC Chaudhari SN Li DK . Bile salt hydrolase activity as a rational target for MASLD therapy. Gut Microbes. (2026) 18:2608437. doi: 10.1080/19490976.2025.2608437 41481156 PMC12773562

[105] Adhikari AA Seegar TCM Ficarro SB McCurry MD Ramachandran D Yao L . Development of a covalent inhibitor of gut bacterial bile salt hydrolases. Nat Chem Biol. (2020) 16:318–26. doi: 10.1038/s41589-020-0467-3 32042200 PMC7036035

[106] Adhikari AA Ramachandran D Chaudhari SN Powell CE Li W McCurry MD . A gut-restricted lithocholic acid analog as an inhibitor of gut bacterial bile salt hydrolases. ACS Chem Biol. (2021) 16:1401–12. doi: 10.1021/acschembio.1c00192 34279901 PMC9013266

[107] Biagioli M Di Giorgio C Massa C Marchianò S Bellini R Bordoni M . Microbial-derived bile acid reverses inflammation in IBD via GPBAR1 agonism and RORγt inverse agonism. Biomedicine Pharmacotherapy. (2024) 181:117731. doi: 10.1016/j.biopha.2024.117731 39657506

[108] Ovadia C Perdones-Montero A Fan HM Mullish BH McDonald JAK Papacleovoulou G . Ursodeoxycholic acid enriches intestinal bile salt hydrolase-expressing Bacteroidetes in cholestatic pregnancy. Sci Rep. (2020) 10:3895. doi: 10.1038/s41598-020-60821-w 32127609 PMC7054423

[109] Gillard J Clerbaux L-A Nachit M Sempoux C Staels B Bindels LB . Bile acids contribute to the development of non-alcoholic steatohepatitis in mice. JHEP Rep. (2022) 4:100387. doi: 10.1016/j.jhepr.2021.100387 34825156 PMC8604813

[110] Martoni CJ Labbé A Ganopolsky JG Prakash S Jones ML . Changes in bile acids, FGF-19 and sterol absorption in response to bile salt hydrolase active L. reuteri NCIMB 30242. Gut Microbes. (2015) 6:57–65. doi: 10.1080/19490976.2015.1005474 25612224 PMC4615650

[111] Lindner S Miltiadous O Ramos RJF Paredes J Kousa AI Dai A . Altered microbial bile acid metabolism exacerbates T cell-driven inflammation during graft-versus-host disease. Nat Microbiol. (2024) 9:614–30. doi: 10.1038/s41564-024-01617-w 38429422 PMC11196888

[112] Cai J Sun L Gonzalez FJ . Gut microbiota-derived bile acids in intestinal immunity, inflammation, and tumorigenesis. Cell Host Microbe. (2022) 30:289–300. doi: 10.1016/j.chom.2022.02.004 35271802 PMC8923532

[113] Li DK Chaudhari SN Lee Y Sojoodi M Adhikari AA Zukerberg L . Inhibition of microbial deconjugation of micellar bile acids protects against intestinal permeability and liver injury. Sci Adv. (2022) 8:eabo2794. doi: 10.1126/sciadv.abo2794 36026454 PMC9417178

[114] Gregor A Auernigg-Haselmaier S Malleier M Bruckberger S Séneca J Pjevac P . Fiber consumption stimulates the activity of microbial bile salt hydrolases. J Funct Foods. (2023) 107:105707. doi: 10.1016/j.jff.2023.105707 38826717

[115] Yang Y Wu C . Targeting gut microbial bile salt hydrolase (BSH) by diet supplements: new insights into dietary modulation of human health. Food Funct. (2022) 13:7409–22. doi: 10.1039/D2FO01252A 35766281

[116] Zöchling A Séneca J Pjevac P Auñon-Lopez A Zebeli Q Pignitter M . Comparative analysis of dietary fiber impact on bile acid metabolism and gut microbiota composition in mice. NPJ Gut Liver. (2025) 2:26. doi: 10.1038/s44355-025-00041-z 41140797 PMC12552122

[117] Wastyk HC Fragiadakis GK Perelman D Dahan D Merrill BD Yu FB . Gut-microbiota-targeted diets modulate human immune status. Cell. (2021) 184:4137–4153.e14. doi: 10.1016/j.cell.2021.06.019 34256014 PMC9020749

[118] Yang C Wan M Xu D Pan D Xia H Yang L . Flaxseed powder attenuates non-alcoholic steatohepatitis via modulation of gut microbiota and bile acid metabolism through gut-liver axis. Int J Mol Sci. (2021) 22:10858. doi: 10.3390/ijms221910858 34639207 PMC8509295

[119] Chiang JYL Ferrell JM . Bile acid receptors FXR and TGR5 signaling in fatty liver diseases and therapy. Am J Physiol Gastrointest Liver Physiol. (2020) 318:G554–73. doi: 10.1152/ajpgi.00223.2019 31984784 PMC7099488

[120] Lai Z Zhan X Lin L Zhang J Qi W Yang H . High-grain diet feeding alters ileal microbiota and disrupts bile acid metabolism in lactating dairy cows. J Anim Sci. (2023) 101:skad278. doi: 10.1093/jas/skad278 37606090 PMC10494876

[121] Xu H Fang F Wu K Song J Li Y Lu X . Gut microbiota-bile acid crosstalk regulates murine lipid metabolism via the intestinal FXR-FGF19 axis in diet-induced humanized dyslipidemia. Microbiome. (2023) 11:262. doi: 10.1186/s40168-023-01709-5 38001551 PMC10675972

[122] Weingarden AR Chen C Bobr A Yao D Lu Y Nelson VM . Microbiota transplantation restores normal fecal bile acid composition in recurrent Clostridium difficile infection. Am J Physiol Gastrointest Liver Physiol. (2014) 306:G310–9. doi: 10.1152/ajpgi.00282.2013 24284963 PMC3920123

[123] Seekatz AM Theriot CM Rao K Chang Y-M Freeman AE Kao JY . Restoration of short chain fatty acid and bile acid metabolism following fecal microbiota transplantation in patients with recurrent Clostridium difficile infection. Anaerobe. (2018) 53:64–73. doi: 10.1016/j.anaerobe.2018.04.001 29654837 PMC6185828

